# Diagnostic challenges of indolent peripheral T cell lymphoma: A case report and literature review

**DOI:** 10.1097/MD.0000000000022657

**Published:** 2020-10-16

**Authors:** Jisoo Lee, Kyeoungseo Park, Kyoung Ha Kim, Hae In Bang, Seug Yun Yoon, In Ho Choi

**Affiliations:** a Soonchunhyang University College of Medicine; b Division of Hematology and Oncology, Department of Internal Medicine, Soonchunhyang University Seoul Hospital; c Department of Laboratory Medicine, Soonchunhyang University Seoul Hospital; d Department of Pathology, Soonchunhyang University Seoul Hospital, Soonchunhyang University College of Medicine, Seoul, Republic of Korea.

**Keywords:** indolent T cell lymphoma, indolent T-cell lymphoproliferative disease of gastrointestinal tracts, low grade T cell lymphoma, peripheral T cell lymphoma

## Abstract

**Introduction:**

Peripheral T cell lymphoma, not otherwise specified (PTCL-NOS) is a heterogeneous group of mature T cell lymphomas which do not correspond to any specific subtype of mature T-cell lymphoma in current classifications. Some researchers have suggested that PTCL with low Ki-67 labeling index should be classified as indolent PTCL

**Patient concerns:**

A 58-year old man diagnosed with alcoholic fatty liver 3 months prior complained of tenesmus and abdominal distension. Colonoscopy of the small and large intestines revealed multiple polyps, which were histologically diagnosed as lymphoid hyperplasia. One month later, he re-visited with a weight loss of 3 to 4 kg over 2 months. Radiologic examination revealed numerous small, homogenous, hypovascular lymph node enlargement in the para-aortic, mesenteric, and both inguinal areas, suggesting malignant lymphoma.

**Diagnosis:**

Laparoscopic biopsy of an omental lymph node was performed, which was histologically confirmed as PTCL-NOS.

**Interventions:**

The patient was administered 3 cycles of cyclophosphamide, doxorubicin, vincristine, and prednisone, but his general condition did not improve. Therefore, treatment was changed to ifosfamide, carboplatin, and etoposide -dexamethasone (4 cycles) followed by allogeneic stem cell transplantation.

**Outcome:**

Even after allogeneic stem cell transplantation, fluorodeoxyglucose uptake in his abdominal lymph nodes and small bowel in positron emission tomography- computed tomography persisted at a Deauville score of 4. The patient has been followed-up for 2 years without progression.

**Conclusion:**

These indolent PTCLs histologically show diffuse infiltrated small lymphoid cells with low KI-67 labeling index and have a relatively good prognosis, although the epidemiology and pathogenesis are not fully elucidated. We report a case of indolent PTCL with cytogenetic abnormalities and poor response to chemotherapy, along with a brief review of the literature.

## 1 Introduction

Peripheral T cell lymphoma, not otherwise specified (PTCL-NOS) is a heterogeneous group of mature T cell lymphomas which do not correspond to any specific subtype of mature T-cell lymphoma in current classifications.^[1]^ Some researchers, especially those in Japan, have suggested that PTCL with low Ki-67 labeling index (Ki-67 LI) should be classified as indolent PTCL.^[2]^ These indolent PTCLs histologically show diffuse infiltrated small lymphoid cells with low KI-67 LI and have a relatively good prognosis, although the epidemiology and pathogenesis are not fully elucidated. We report a case of indolent PTCL with cytogenetic abnormalities and poor response to chemotherapy, along with a brief review of the literature.

## 2 Case description

A 58-year old man diagnosed with alcoholic fatty liver 3 months prior complained of tenesmus and abdominal distension. Colonoscopy of the small and large intestines revealed multiple polyps, which were removed. Histologic examination showed lymphoid hyperplasia in both the small and large intestinal polyps. Complete blood count indicated lymphocytosis (white blood cell count 5.4 × 103/μL, hemoglobin 15.3 g/dL, platelet count 182 × 103/μL, 16.68% neutrophils, and 75.09% lymphocytes) as well as unremarkable chemistry findings.

**Figure 1:**
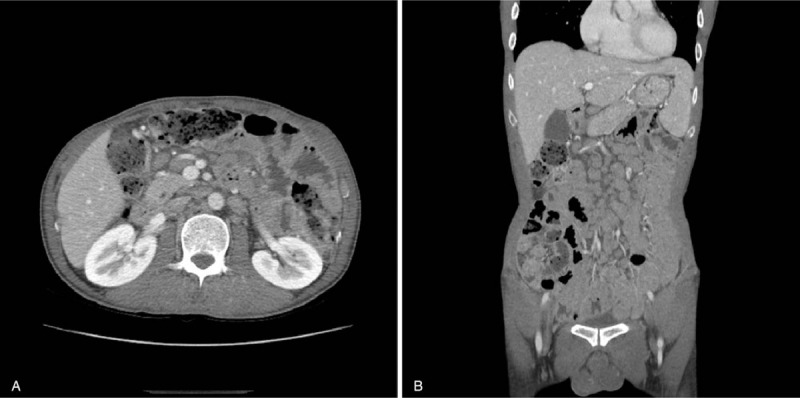
Contrast-enhanced abdominopelvic axial (A) and coronal (B) computed tomography (CT) scans of the case. (A-B) Numerous, small, homogenous, hypovascular lymph nodal enlargement visible in the paraaortic, mesenteric, and both inguinal areas.

**Figure 2:**
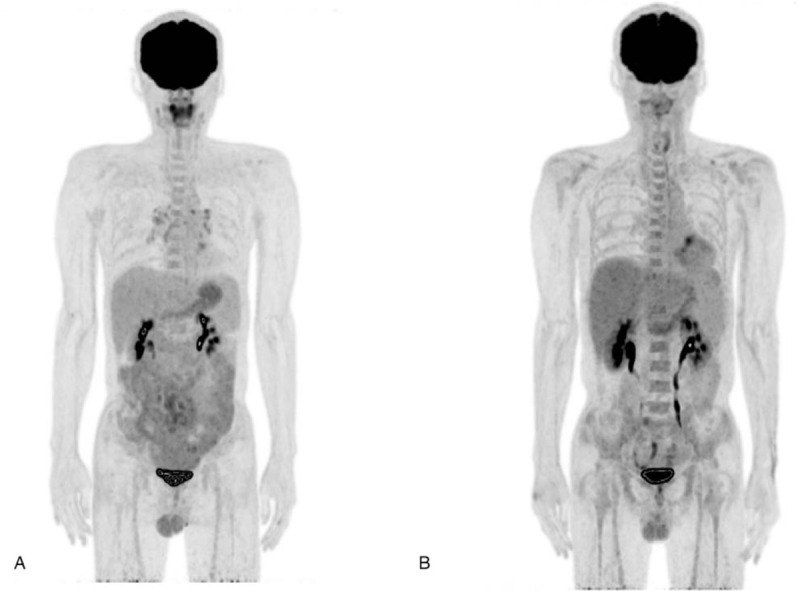
Positron emission tomography-computed tomography (PET-CT) findings. (A) Mild diffuse FDG uptake along nearly the entire small bowel (Deauville score 4) and mildly hypermetabolic lymph nodes in the bilateral neck, bilateral mediastinum, mesentery, and retroperitoneum (SUVmax = 3.8, Deauville score 4). (B) PET-CT findings after chemotherapy and allogeneic bone marrow transplantation (BMT). The image shows slightly decreased FDG uptake with residual malignant involvement in the abdominal lymph nodes (Deauville score 4), small bowel (Deauville score 4), bone marrow and sigmoid colon (Deauville score 3), and lymph nodes in mesentery and retroperitoneum (SUVmax = 1.1, Deauville score 2). FDG = fluorodeoxyglucose.

One month later, he re-visited with a weight loss of 3 to 4 kg over 2 months. Contrast-enhanced abdominopelvic computed tomography (CT) revealed numerous small, homogenous, hypovascular lymph node enlargement in the para-aortic, mesenteric, and both inguinal areas (Fig. 1). Positron emission tomography-CT (PET-CT) performed for suspected malignant lymphoma showed a mild diffuse increase in fluorodeoxyglucose uptake throughout the small intestine (Deauville score 4, Fig. 2A). There were also mildly hypermetabolic lymph nodes in the bilateral neck, bilateral mediastinum, mesentery, and retroperitoneum, suggesting a low-grade malignant lymphoma. Laparoscopic biopsy of an omental lymph node was performed for diagnostic confirmation.

**Figure 3:**
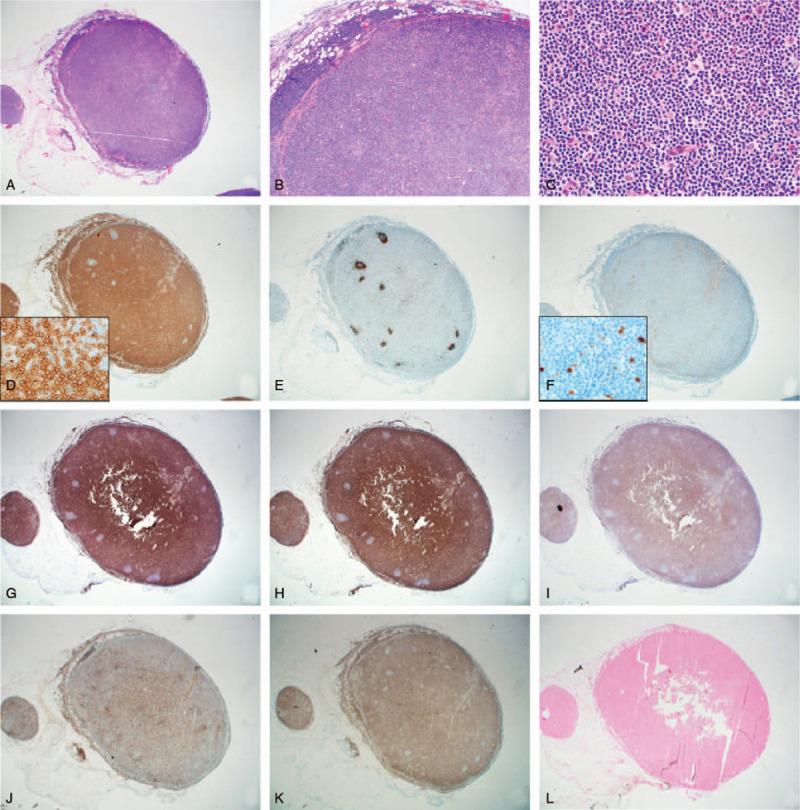
Morphological and immunohistochemical features of an omental lymph node. (A) Biopsy specimen showing diffuse effacement of the normal lymph node architecture (hematoxylin-eosin [H&E], 12.5 × ). (B) Diffuse architectural effacement with lymphoid infiltration of the perinodal fatty tissue. (H&E, × 40). (C) Tumor cells predominantly consisting of small lymphocytes with no or mild atypia (H&E, x400) (D-E) Tumor cells predominantly expressing CD3 (D), with residual primary follicles composed of CD20-positive cells at the periphery (E). (F) Very low Ki-67 LI (less than 5%). (G-I) Tumor cells positive for CD2 (G) and CD5 (H) and weakly positive for CD7 (I). (J-K) While some T cells are positive for CD4 (J), most were positive for CD8 (K). (L) Epstein–Barr virus (EBV) in situ hybridization shows negative result.

Microscopically, the omental node showed diffuse architectural effacement or para-follicular expansion in low-power views (Fig. 3A and B). The effaced area consisted mainly of small lymphocytes with no or mild cellular atypia. The cells were positive for CD3, with some residual primary follicles composed of CD20-positive cells remaining at the periphery (Fig. 3C–E). Overall, the Ki-67 LI was less than 5%. (Fig. 3F) Other immunohistochemical results included CD4(+) in a minority of T cells, CD8(+) in the majority of T cells, CD2(+), CD5(+), CD7(+, weak), CD10(−), CD21(−), CD56(−), CD30(−), and ALK(−) (Fig. 3G–K). Epstein–Barr virus (EBV) in situ hybridization was negative (Fig. 3I). Although the Ki-67 LI was low, the histological, immunohistochemical, and clinico-radiological findings were consistent with malignant T cell lymphoma, confirming PTCL-NOS.

**Figure 4:**
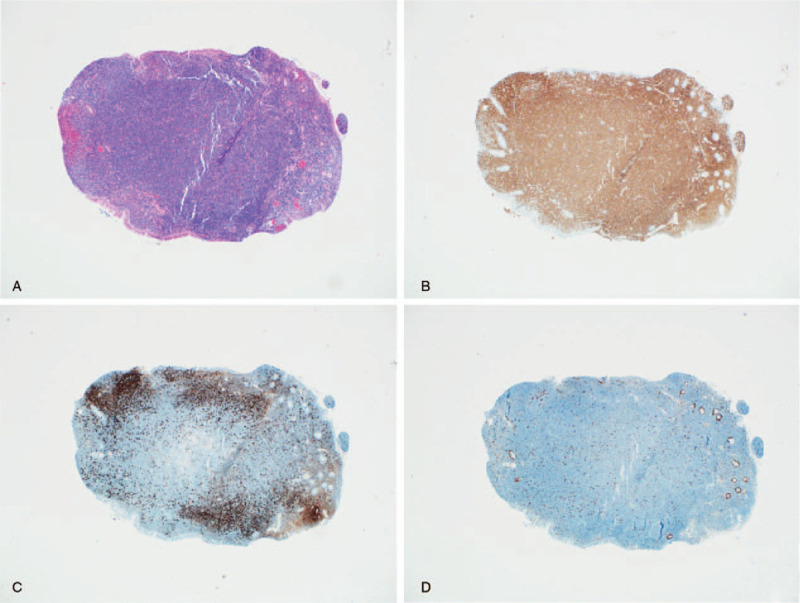
Terminal ileum biopsy performed 1 month before diagnosis. (A) Marked lymphoid infiltration in the endoscopic biopsy of the terminal ileum without remarkable germinal centers. (H&E, x40) (B) Infiltrated lymphoid cells mainly expressing CD3. (C) Residual CD20-positive cells near the lining epithelium. (D) Low Ki-67 LI.

**Figure 5:**
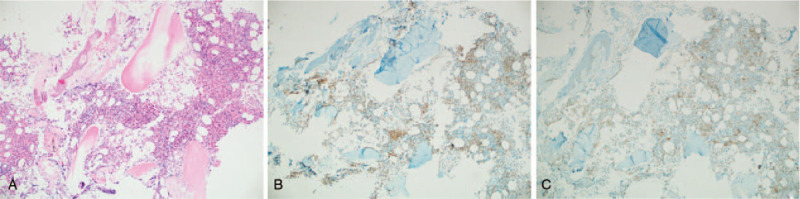
Findings of bone marrow biopsy. (A) Multiple small lymphocyte aggregates in the interstitium (A, H&E, x100). (B,C) Infiltrated lymphocytes are positive for CD3 (B) and CD8 (C).

**Figure 6:**
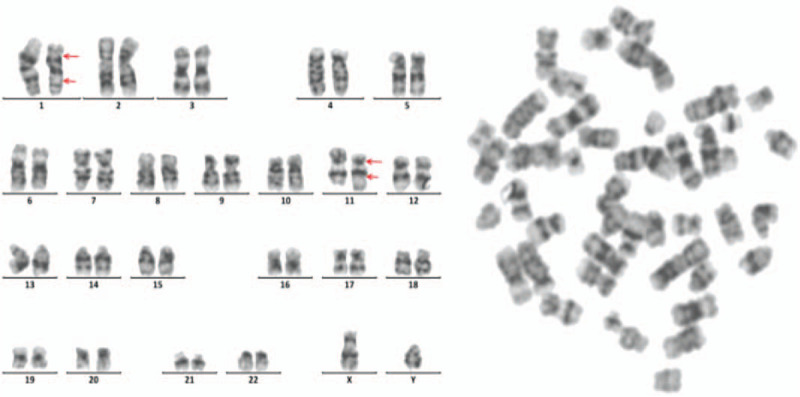
Chromosome analysis. G-band method showing clonal chromosomal abnormalities in 4 of 30 cells. Sequences of unknown origin were added and deleted on the short arm of chromosome 1 and added on chromosome 11 (46,XY,der(1)add(1)(p32)del(1)(q25q32),der(11)add(11)(p11.2)add(11)(q23)).

The previous biopsies of the patient's colon and small intestine were also reviewed, which showed diffuse infiltration of small lymphoid cells consisting mainly of small CD3(+)cells with a low Ki-67 LI (Fig. 4). Bone marrow biopsy showed multiple lymphocyte aggregates in the interstitium, suggesting T cell lymphoma (Fig. 5). Flow cytometric analysis of the bone marrow aspirate revealed small to medium-sized lymphocytes positive for CD2, CD3, CD5, CD7, and a decreased CD4:CD8 ratio of 0.1. Conventional bone marrow chromosome analysis showed 46,XY,der(1)add(1)(p32)del(1)(q25q32),der(11)add(11)(p11.2)add(11)(q23) in 4 of 30 metaphase cells (Fig. 6).

Under the diagnosis of PTCL with stage IV, the patient was administered 3 cycles of cyclophosphamide, doxorubicin, vincristine, and prednisone, but his general condition did not improve. Therefore, treatment was changed to ifosfamide, carboplatin, and etoposide -dexamethasone (4 cycles) followed by allogeneic stem cell transplantation (SCT). On the PET-CT taken 1 month after SCT, fluorodeoxyglucose uptake in his abdominal lymph nodes and small bowel persisted at a Deauville score of 4 (Fig. 2B), and it was thought to be a poor response. However, subsequent chest and abdominopelvic CT during 2 years after SCT, no disease progression has been noted.

The Institutional Review Board of Soonchunhyang university Seoul hospital approved reporting of this case (IRB File no: SCHUH 2019-10-002). Patient has provided informed consent for publication of the case.

## 3 Discussion

PTCL-NOS, a heterogeneous group of mature T cell lymphomas that do not fit into any of the other subtypes of PTCL, accounts for nearly 30% of PTCL. Histologically, most cases of PTCL-NOS show numerous medium to large-sized cells with irregular, pleomorphic, hyperchromatic nuclei with frequent mitoses. However, some cases with monomorphic small neoplastic infiltrate and low Ki-67 proliferative index have been observed, which seem to be associated with an indolent course and may undergo spontaneous regression without therapy. Therefore, some researchers have proposed classifying these cases as indolent PTCL (low-grade PTCL).^[2]^

To our knowledge, 22 cases of indolent PTCL with low Ki-67 LI have been reported in the literature, which are summarized in Table 1 .^[2–6]^ These 22 cases included 11 men and 11 women (M: F = 1: 1) with an average age of 53.5 years (range: 15–82). All cases showed diffuse infiltrate of small to medium-sized lymphocytes with low Ki-67 LI (less than 10%). PTCL containing only lymph nodes was reported in 3 patients; the other patients (19/22, 86.4%) had extranodal involvement including 11 from the gastrointestinal tract (GIT) (50.0%), 4 from the spleen (18.2%), and 4 from the thyroid (18.2%). Immunohistochemical staining revealed a slight predominance of the CD4(−)/CD8(+) type over CD4(+)/CD8(−) (13 vs 9 cases) but there was no significant difference according to the involved sites. Except for 2 cases in which the treatment method was not reported, nine patients were administered only chemotherapy, 3 received both surgical resection and chemotherapy, 3 underwent only surgical resection, and 5 received conservative treatment including steroid therapy. Two of the 22 patients died; patient No.10 due to autoimmune hemolytic anemia during steroid monotherapy and patient No.19 due to sepsis induced by small intestinal perforation 11 years after the initial diagnosis. Most other patients had an indolent clinical course in which the disease subsided, or the patient lived with the disease at follow-up.

**Table 1:**
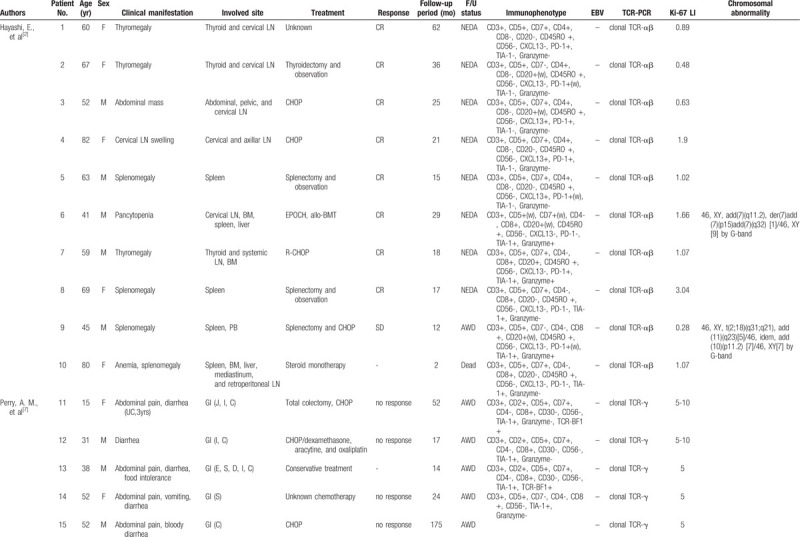
Review of literature of indolent T-cell lymphoma showing low Ki-67 LI.

**Table 1 (Continued):**
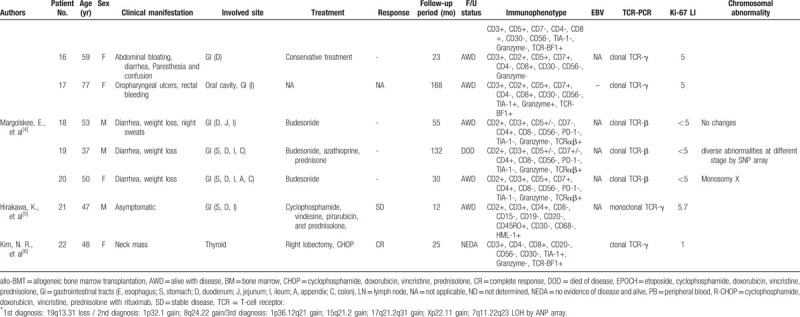
Review of literature of indolent T-cell lymphoma showing low Ki-67 LI.

Considering the cases with complete remission following surgical resection, cases with poor response after chemotherapy but without aggressive disease progression, and alive cases with conservative treatment, these low-grade histological findings raise doubts about the diagnosis of malignant T-cell lymphoma. However, the diagnosis of lymphoma might not be problematic because all reviewed cases were monoclonal by T-cell receptor-polymerase chain reaction or Southern blot.

One peculiarity is that the extranodal involvement sites in the literature are organs that frequently develop extranodal marginal zone lymphoma of mucosa-associated tissue (MALToma), which is considered a low-grade B-cell lymphoma. T cell lymphomas occurring in the GIT are generally rarer than the B cell types and are aggressive. Several cases of T-cell lymphoproliferative disease with low Ki-67 LI and indolent clinical course have been reported recently but their correlation with MALToma seems requires further study.^[4,7]^ Except for indolent T-cell lymphoma in the GIT, Table 1  includes 4 thyroid cases. In a study by Yoshida et al (not included in Table 1  because it did not include Ki-67 LI), primary thyroid T-cell lymphoma (PTTL) also showed an indolent clinical course.^[8]^ The authors suggested that the PTTL of Th1-cell origin might be a T-cell malignancy corresponding to a MALToma and that PTTL is a distinct T-cell neoplasm among PTCL-NOS because autoimmune thyroiditis is associated with development with MALToma by mediation of Th1-cells. Kim et al reported lymphoepithelial lesion-like changes in a review of thyroid T cell lymphomas and suggested that the close association between follicular cells and neoplastic lymphocytes simulating lymphoepithelial lesions of MALTomas may be characteristic of PTTL.^[6]^ Further study is needed on indolent PTCL in a background of autoimmune thyroiditis.

Indolent PTCL is not easily diagnosed from histological and immunohistochemical findings. The parafollicular expansion seen in T-cell lymphomas is also seen in reactive lymphadenopathy caused by various causes including infectious diseases; thus, differential diagnosis depends on cellular atypia of tumor cells and high KI-67 LI. In the present case, although the diagnosis of omental lymph node was not easy, review of the previous endoscopic biopsy slides showed that the diagnosis of malignant T-cell lymphoma was histologically difficult to confirm unless there was PET-CT evidence to suggest lymphoma.

The cutoff value of Ki-67 may be a sensitive issue in the diagnosis of indolent PTCL. Generally, lymphomas with higher Ki-67 LI are considered to be more aggressive. One study showed that indolent non-Hodgkin lymphoma (NHL) had low Ki-67 LI (less than about 30%), including 23% for small cell lymphoma, 25%, for mantle cell lymphoma, 28.5% for marginal zone lymphoma, and 34.6% for follicular lymphoma, whereas the mean Ki-67 LIs of aggressive lymphomas were above 66.4%.^[9]^ However, this observation mainly reflects data from B-cell lymphomas. Hayashi et al favored 5% as the cutoff value for indolent PTCL,^[2]^ while Perry et al used 10% as the cutoff for indolent T-cell lymphoproliferative disease of GIT.^[7]^ Therefore, establishing the entity of indolent T-cell lymphoma as well as the criteria for Ki-67 LI, are required.

In conclusion, we reported a case of low-grade PTCL with poor response to chemotherapy, which was missed at the initial colonoscopic biopsy due to its low-grade histologic features indistinguishable from lymphoid hyperplasia. Although indolent PTCL is not yet an established entity in the WHO classification of T-cell lymphoma, gathering of these case reports may be helpful for further treatment and studies of T-cell lymphomas.

## Author contributions

**Conceptualization:** In Ho Choi.

**Data curation:** Kyoung Ha Kim.

**Methodology:** Hae In Bang.

**Supervision:** Seug Yun Yoon, In Ho Choi.

**Writing – original draft:** Jisoo Lee, In Ho Choi.

**Writing – review & editing:** Kyeoungseo Park, Kyoung Ha Kim, Hae In Bang.
